# Social and behavior change communication competency among front-line healthcare system actors in Ethiopia: a cross-sectional study

**DOI:** 10.1186/s12889-024-18084-x

**Published:** 2024-03-01

**Authors:** Simegnew Handebo, Rachana Sharma, Tesfaye Simireta, Hailemariam Addissie, Getnet Bayih Endalew, Eshetu Girma, Kenzudin Assfa Mossa

**Affiliations:** 1 School of Public Health, Saint Paul’s Hospital Millennium Medical College, Addis Ababa, Ethiopia; 2 Ethiopian Health Education and Promotion Professionals Association (EHEPA), Addis Ababa, Ethiopia; 3 UNICEF, Addis Ababa, Ethiopia; 4 Former SBC Specialist, UNICEF, Addis Ababa, Ethiopia; 5 Health Education and Promotion Team Lead, Federal Ministry of Health, Addis Ababa, Ethiopia; 6 Technical Lead-Risk Communication and Community Engagement, WHO-Ethiopia, Addis Ababa, Ethiopia; 7 Department of Preventive Medicine, School of Public Health, Addis Ababa University, Addis Ababa, Ethiopia; 8 Department of Public Health, College of Medicine and Health Sciences, Wolkite University, Welkite, Ethiopia

**Keywords:** Front-line health actors, SBCC competencies, Knowledge, Skills, Ethiopia

## Abstract

**Introduction:**

Social and Behavior Change Communication (SBCC) plays a critical role in improving behavior and health outcomes across the continuum of healthcare. Failing to implement tailored SBCC strategies continues to pose a risk of ill health, increase disease burden, and impact the quality life of people. In Ethiopia, front-line healthcare system actors’ knowledge and skills about SBCC have not been rigorously assessed. Thus, the current study aimed to assess healthcare system actors’ competencies in designing, implementing, monitoring, and evaluating SBCC interventions in Ethiopia.

**Methods:**

A cross-sectional study was conducted between 01 August and 31 October, 2020. Five hundred twenty-eight frontline healthcare system actors in SBCC in Ethiopia were included using simple random sampling technique. Data was collected using a self-administered structured questionnaire adopted from Communication for Change; SBCC capacity assessment tool. Descriptive analysis frequencies, percentages, mean, median, standard deviation (SD), interquartile range (IQR) were employed. Besides correlations and linear regression with robust standard errors were carried out. A 95% confidence interval and a *p*-value of less than 0.05 were used to declare significant statistical association.

**Results:**

A total of 488 frontline workers participated in the study, with a response rate of 92.4%. The mean SBCC knowledge score was 13.2 ± standard deviation (SD) 3.99 and 59.2% scored below 60% of the expected maximum score. The standard mean score of overall skill in SBCC intervention was 2.36 (SD ± 0.98) and 52.6% of them scored below mean score. The SBCC knowledge was significantly predicted by the service year and the regional variation. On the other hand, SBCC skills was significantly predicted by sex, service year, profession, regional variation, and SBCC knowledge. The regional variation was the main predictor of both knowledge and skill on SBCC. The regression models explained 23.1% and 50.2% of the variance in knowledge and skill of SBCC, respectively.

**Conclusion:**

Front-line healthcare system actors in Ethiopia has low knowledge and skills in SBCC. Variations in SBCC knowledge and skill were observed based on demographic and professionals experience related characteristics. Hence, continuous capacity building activities need to be given to frontline healthcare system actors to enhance their knowledge and skill on SBCC program and achieve the intended health results.

**Supplementary Information:**

The online version contains supplementary material available at 10.1186/s12889-024-18084-x.

## Introduction

Health is a foundation for people’s comprehensive development and well-being. It is a precondition for the social, economic, and environmental dimensions of sustainable development goals (SDGs). Integrating health promotion into change efforts has the potential to advance and multiply the SDGs. There is a mutually reinforcing relationship and overlap between health promotion and the SDGs [1]. Health promotion has a significant role to play in reducing the burden of disease to the health system, by addressing the key social, behavioral and structural determinants of health [2]. Communication is a powerful tool to address growing and complex health challenges [3]. In the 21st century, with ever-increasing public health threats and challenges, there is an increasing demand for innovative health communication approaches for behavioral changes to alleviate public health problems at large [4].

People’s behaviors often cannot be achieved without shaping the social and environmental factors that influence their behaviors [5–7]. This informs the need for SBCC interventions like providing tailored messages and a supportive environment that persuades individuals and communities to make positive health behavior changes [8]. SBCC is the process of modifying or maintaining healthy behaviors, improving health services, and enhancing health outcomes at the individual, community, and social levels by strengthening the social context, systems, and processes using theoretically, culturally, and contextually appropriate approaches. SBCC is premised on the assumption that individual behaviors need to be understood and tackled within an ecological framework, from interpersonal relations to the policies, cultural norms, and values that shape the world in which individuals live [9–11]. SBCC plays an important role in introducing and maintaining desired health behaviors and norms [12].

In Ethiopia, SBCC is mainly carried out by frontline healthcare system actors including health extension workers (HEWs) as their programs highly focused on prevention of health problems and enhancing healthcare services utilization at the community level [13–15]. It is expected of community health workers to dedicate an average of 10% of their time to health promotion programs in order to meet community needs and support state-wide initiatives [16]. Despite remarkable achievements in health service in country, there are still significant gaps in the health status of the population. The SBCC is highlighted and will remain a key component of the Health Sector Transformation Plan (HSTP) in Ethiopia for enhancing community empowerment and positive health outcomes [17]. This require reorienting the health services towards health promotion and increase in the capacity of the health service staff [16, 18]. In 2016, Ethiopia developed a National Health Promotion and Communication Strategy to provide a framework to support specific SBCC interventions [19].

The evolution and expansion of health promotion over the years has demanded human resources with specific skills and competencies to effectively implement health promotion interventions like [20]. Thus, having adequate numbers and a mix of motivated and skilled human resources in SBCC areas is essential at all levels of the healthcare system [21]. A study also found that health professionals who are not usually associated with health promotion practices are knowledgeable and wish to focus more on health promotion and disease prevention [18]. The Ethiopian HSTP emphasized on the skill and knowledge of healthcare providers and health communication professionals to provide quality health services and information [17].

The frontline healthcare actors’ knowledge and skills on SBCC is critical for successful SBCC program design, implementation, monitoring and evaluation. It is also important to identify what key capacities already exist and what additional capacities may be needed to reach SBCC program objectives [22]. In Ethiopia, the capacity of frontline healthcare system actors on SBCC interventions has not been assessed. Therefore, this study was aimed at assessing the frontline healthcare system actors’ capacities in SBCC intervention design, implementation, monitoring and evaluation in Ethiopia.

## Methods

### Study design, setting, and population

A cross-sectional study was conducted among frontline health actors in Ethiopia between 01 August and 31 October, 2020. Ethiopia is a landlocked country in the Horn of Africa. As of 2020, it is home to around 113.5 million inhabitants, making it the 12th-most populous country in the world and the 2nd-most populous in Africa after Nigeria [23, 24]. The national capital and largest city is Addis Ababa. Ethiopia experiences public health problems typical of an underdeveloped country, such as communicable diseases, maternal and child health problems and malnutrition; these account for the majority of public health problems. Non-communicable diseases and injuries are also growing. In 2020, 367 public hospitals, 3,777 health centers and 17,699 health posts were providing services in the country. Regarding the health workforce capacity of the sector, 212,185 healthcare workers are providing health services in public health institutions including Nurses (69,824), Health Extension workers (42,630) and Midwifery (20,355) [25]. Front-line healthcare system actors for SBCC including Health Extension Workers (HEWs) and other health workers participating in SBCC activities in Ethiopia were the source population of the study. Selected front-line healthcare system actors working on SBCC activities in selected regions and Addis Ababa were the study population of the study.

### Sample size and sampling procedure

The sample size was determined by using a single population proportion formula with the following statistical assumptions: the proportion of front-line healthcare system actors with a high level of overall knowledge of SBCC was 50% (since there was no similar previous study done on the area); Zα/2 (the value of the standard normal curve score corresponding to the given confidence interval = 1.96) corresponding to a 95% confidence level; d (degree of desired precision at = 5%), a design effect of 1.25, and a non-response rate of 10%. The final sample size was 528.

Four regions (Oromia, Amhara, SNNPR, and Gambella) and one city administration (Addis Ababa) were selected randomly and included in this study. First, participants from each region were stratified into urban and rural settings. Then, the rural setting was further stratified as agrarian and pastoralist. Representatives from urban and rural (agrarian and pastoralist) settings and from all programmatic areas of regions were included. The total sample size was allocated to each region proportionally based on the number of front-line healthcare system actors for SBCC in the regions. The sampling frame was developed by listing front-line healthcare system actors for SBCC at the district level. A simple random sampling technique was used to draw participants from randomly selected districts.

### Data collection tools and procedures

The data were collected using a self-administered structured questionnaire adopted from the C-Change; social and behavior change communication capacity assessment tool [26]. The tool has socio-demographic characteristics, and knowledge and skills based on five domains: Understanding the Context through Situation Analysis, Focusing & Designing the Communication Strategy, Creating Interventions & Materials for Change, Implementing & Monitoring Change Processes, and Evaluating & Re-planning the Program. Experienced data collectors/facilitators who had at least a Bachelor of Science in public health were used for the process of data collection. Data collectors and supervisors were given training on the objective of the study, the technique of data collection, the content of the questionnaire, and ethical issues they needed to take into account during data collection.

### Study variables

The outcome variable of this study was the SBCC competencies of front-line healthcare system actors. The independent variables were socio-demographic variables (age, sex, residence, and region), profession, service year, training on SBCC, and knowledge of SBCC.

### Operational definition

#### Knowledge of SBCC

Knowledge of SBCC measures one’s current cognition level around various steps of SBCC program design, implementation, and evaluation. It was measured with 26 items in the five-step process called C‐Planning and recoded as one for correct responses and zero for incorrect responses [11]. Cronbach’s alpha reliability test was 0.715. Responses to the questions were added together to generate an overall knowledge score. The composite score ranges from zero to twenty-six and the higher score indicates higher knowledge of SBCC among individuals. For descriptive statistics purpose the composite knowledge score was categorized using Bloom’s cut-off point, as low-level of knowledge (less than 60%), moderate level of knowledge (60–80%) and good level of knowledge (80–100%).

#### SBCC related skill

SBCC-related skills measure a person’s demonstrated expertise and skills needed to manage, implement, and evaluate SBCC programs. It was measured using 26 items on a five-point scale in a five-step process called C-Planning. Skill questions were analyzed based on the classification as follows: 1 = No skill/competency (if the participant has never performed a given competency and does not know how); 2 = Novice level (if the participant has a superficial understanding of a given competency but not enough to perform it); 3 = Apprentice level (if the participant can perform a given competency but could use some additional training on it); 4 = Professional level (if the participant can perform a given competency well and apply it in his/her work); and 5 = Expert level (if the participant can perform a given competency, apply it, and train others in it). Responses to the questions were added together to generate an overall skill score [11]. The composite score ranges from twenty-six to one hundred thirty and the higher score indicates advanced skill in SBCC.

##### SBCC competency

Is knowledge and skills on SBCC, systematic application of interactive, theory-based and research-driven communication processes and strategies to address change at individual, community, and societal levels. It was measured as ‘knowledge of SBCC’ and ‘SBCC related skill’ separately adopting SBCC Capacity Assessment Tools.

##### Frontline healthcare system actors for SBCC

are healthcare workers (e.g., nurses, health officers, and HEWs) who are working on the frontlines with clients on a regular basis and apply SBCC knowledge and skill to deliver higher quality care to clients.

### Data processing and analysis

The data were cleaned, coded, and analyzed using Stata version 14. The skewness and kurtosis tests [(for knowledge: -0.23 and 2.42, respectively) and (for skill: 0.48 and 2.65, respectively)] were used to verify the outcome variable’s normality assumptions, and their distribution was slightly skewed. However, when the sample size is more than 300, the skewness between + 2 and − 2 is regarded as normal [27]. Thus, simple linear regression was used to examine the association between the independent variable and knowledge and skill in SBCC. The assumptions of linearity, normality, constant variance, outliers, and multi-collinearity were examined. To test the linearity assumptions, a scatter plot was employed, and the variables revealed linear relationships.

In order to determine homoscedasticity, a Cameron and Trivedi’s decomposition of the IM-test was performed; however, because the *p*-value was higher than 5%, there was no proof of the existence of heteroscedasticity. The variance inflation factor (VIF), used to test for multi-collinearity, revealed that all variables had values lower than five. The Cook’s D test showed the presence of outliers and due to this, robust regression were carried out to account for the assumptions. The amount of variation in knowledge and skill in SBCC explained by the independent variables was evaluated using R-square. An unstandardized β coefficient was used to interpret the effect of the independent variables.

## Results

### Socio-demographic characteristics of study participants

A total of 488 front-line healthcare system actors participated in this study, with a response rate of 92.4%. The non-response was due to not replying to items on the questionnaire (3.1%), inconsistent responses (2.0%) and not volunteered without reason (2.5%). Nearly two-thirds (64.5%) of the study participants were female. Nearly one-fourth (24.0%) of caregivers were aged below 30 years. The median age of respondents was 29 years, with an inter-quartile range of 7 years. Nearly four of the ten participants were 25–29 years old. More than half (57.4%) of the participants were from urban areas. About a quarter (23.2%) of healthcare system actors were from the Oromia region. Of the participants, 27.5% had experiences ranging eight to eleven years, and 39.5% were nurses (Table 1).

**Table 1:** Socio-demographic characteristics of frontline healthcare system actors in Ethiopia, 2020

Variable	Categories	Frequency (n)	Percentage (%)
Sex	Male	173	35.5
	Female	315	64.5
Age	≤ 24 years	54	11.1
	25–29 years	192	39.3
	30–34 years	135	27.7
	≥ 35 years	107	21.9
Place of residence	Urban	280	57.4
	Rural agrarian	111	22.7
	Rural pastoral	97	19.9
Region	Oromia	113	23.2
	Addis Ababa	106	21.7
	Amhara	102	20.9
	SNNPR	94	19.3
	Gambella	73	15.0
Service year	1–4 years	127	26.0
	5–7 years	120	24.6
	8–11 years	134	27.5
	≥ 12 years	107	21.9
Profession	Nurse	193	39.5
	HEW	88	18.0
	Health officer	79	16.2
	Midwife	31	6.4
	Others*	97	19.9

*: Medicine, environmental health, health education, masters of public health and social science studies

### Healthcare system actors’ knowledge of SBCC

From the study participants, 35.5% reported that they had taken at least one SBCC or related health communication training in the last five years. The mean overall SBCC knowledge score was about 13.2, with a standard deviation of 3.99. Of the sub-domains, the highest mean score was observed on understanding the situation (mean = 2.9 ± SD1.22) and the lowest was on creating interventions and materials for behavior change (mean = 1.40 ± SD 0.94) (Table 2). The item level knowledge score was provided in Supplementary file 1.

**Table 2:** Descriptive statistics of SBCC knowledge sub-domains among frontline healthcare system actors in Ethiopia, 2020

Variables	Number of items	Mean	SD	Median	IQR	Min	Max
Overall SBCC knowledge	26	13.20	3.99	14	6	2	22
Basic SBCC concepts	3	2.00	0.93	2	2	0	3
Understanding the situation	5	2.92	1.22	3	2	0	5
Focusing and designing the communication strategy	5	2.36	1.25	2	2	0	5
Creating interventions and materials for change	4	1.40	0.94	1	1	0	4
Implementing and Monitoring the Change Process	4	2.18	0.99	2	1	0	4
Evaluating and Re-planning	5	2.32	1.25	2	1	0	5

SD: Standard Deviation; IQR: Interquartile Range; Min: Minimum; Max: Maximum

**Fig. 1:**
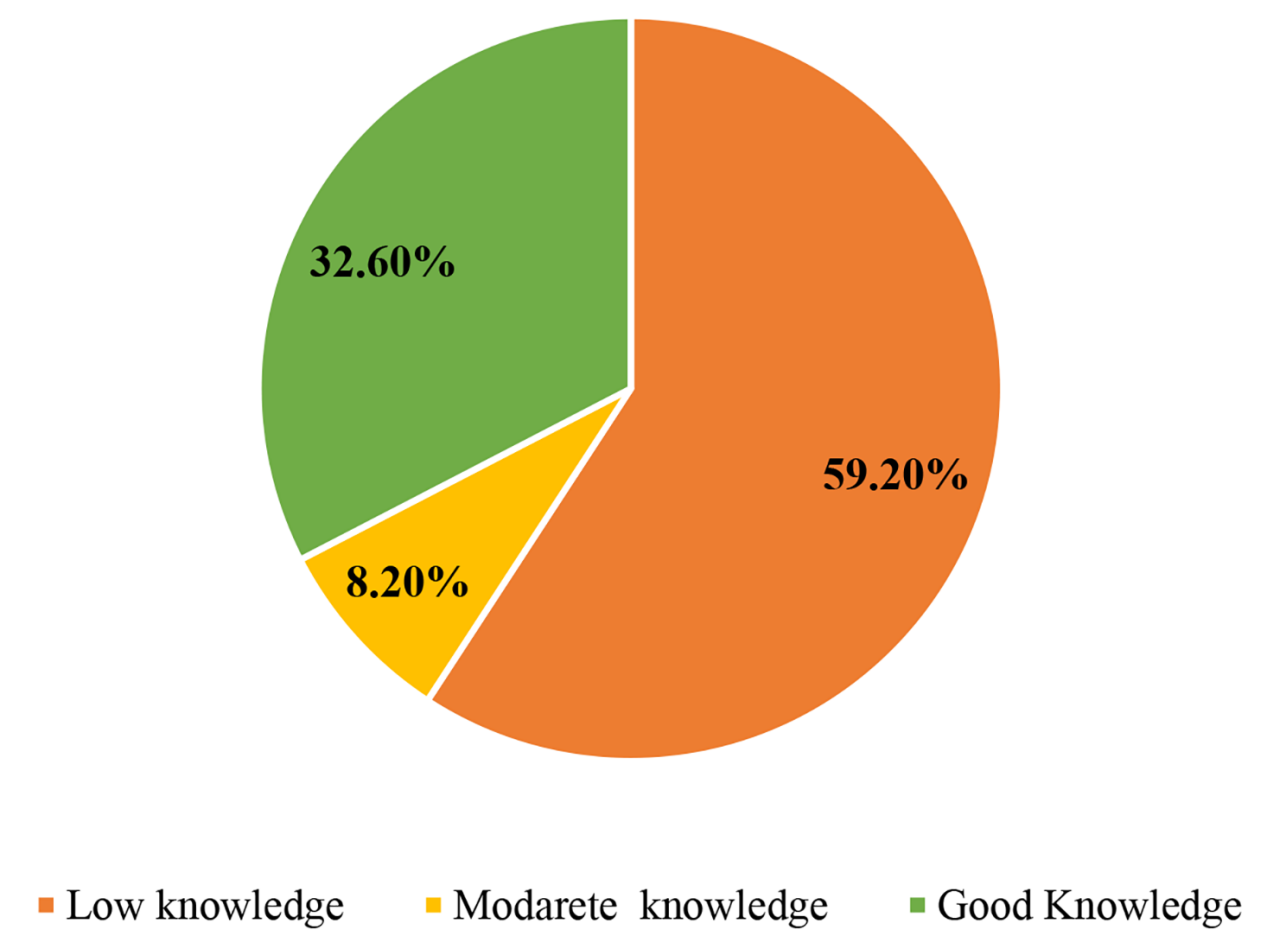
Overall knowledge of SBCC among frontline health system practitioners in Ethiopia, 2020

Based on Bloom’s classification of knowledge more than half (59.2%) of the participants scored below 60% of the sum score and had poor knowledge, while only one third (32.6%) of them had good knowledge (Fig. 1).

### Correlation among sub-domains of SBCC knowledge

A spearman rank correlation analysis was done to explore the relationship among the sub-domains of SBCC knowledge. Accordingly, most sub-domains of SBCC knowledge showed a week positive and significant correlation among each other at a *p*-value of < 0.05. However, there was no significant correlation between the basic SBCC concept and creating interventions and materials for change domains, as well as understanding the situation and evaluating and re-planning sub-domains. The overall SBCC knowledge has a moderate and positive association with the sub-domain of SBCC knowledge at a *p*-value of < 0.05 (Table 3).

**Table 3:** Spearman rank correlation of SBCC knowledge sub-domains among front line healthcare system actors in Ethiopia, 2020

Variables	1	2	3	4	5	6	7
1. Overall knowledge	1						
2. Basic SBCC concepts	0.51*	1					
3. Understanding the situation	0.53*	0.12*	1				
4. Focusing and designing the communication strategy	0.69*	0.21*	0.23*	1			
5. Creating interventions and materials for change	0.49*	0.06	0.25*	0.20*	1		
6. Implementing and monitoring the change process	0.61*	0.31*	0.17*	0.29*	0.19*	1	
7. Evaluating and re-planning	0.67*	0.28*	0.09	0.40*	0.17*	0.36*	1

*= Correlation is significant at 0.05(2 tailed)

### Predictors of SBCC knowledge

Place of residence, sex, regional states, age, service year, profession, and SBCC related training were candidates for multiple linear regression. In multiple linear regression variables statistically significant at a 5% significance level were service year and regional state. The standardized regression coefficient suggested that being from Gambella region (β=-0.51) was the main predictor of knowledge of SBCC, followed by being from SNNPR (β=-0.25) and Amhara region (β= -0.24). The variance explained in SBCC knowledge from all predictors was 23.1%.

Frontline health workers who had an experience of 5–7 years (β = 1.71, 95% CI (0.65, 2.78)), 8–11 years (β = 1.24, 95% CI (0.09, 2.39)), and above twelve years (β = 1.78, 95% CI (0.45, 3.11)) had 1.71, 1.24, and 1.78 times increased knowledge of SBCC compared to those who had less than or equal to four years of experience, respectively holding other variables constant. Similarly, frontline health workers from Amhara (β=-2.38, 95% CI (-3.64, -1.13)), SNNPR (β=-2.49, 95% CI ((-4.00, -0.99)), and Gambella (β=-5.75, 95% CI (-8.15, -3.36)) regions had 2.38, 2.49, and 5.75 decreased knowledge of SBCC than those from Addis Ababa city, holding other variables constant (Table 4).

**Table 4:** Multiple linear regression analysis result of SBCC knowledge among frontline healthcare system actors in Ethiopia, 2020

Variables		Unstandardized beta(B)	Standardized beta (β)	95% CI of B
Constant		12.56		(10.89, 14.24)
Sex	Female (ref)			
	Male	0.43	0.05	(-0.31, 1.16)
Residence	Urban (ref)			
	Rural agrarian	0.49	0.05	(-0.32, 1.29)
	Rural pastoralist	1.14	0.11	(-0.89, 3.17)
Age	≤ 24 years (ref)			
	25–29 years	0.44	0.05	(-0.88, 1.76)
	30–34 years	0.89	0.10	(-0.61, 2.39)
	≥ 35 years	0.62	0.06	(-0.97, 2.21)
Service year	1–4 years (ref)			
	5–7 years	1.71	0.18	(0.65, 2.78)*
	8–11 years	1.24	0.14	(0.09, 2.39)*
	≥ 12 years	1.78	0.18	(0.45, 3.11)*
Profession	Nurse (ref)			
	HEW	0.73	0.07	(-0.30, 1.77)
	Health officer	-0.04	-0.004	(-1.34, 1.26)
	Midwife	-0.19	-0.01	(-1.55, 1.16)
	Others	0.47	0.04	(-0.32, 1.27)
Ever took SBCC related trainings	No (ref)			
	Yes	0.61	0.07	(-0.28, 1.49)
Region	Addis Ababa (ref)			
	Oromia	-0.60	-0.06	(-1.77, 0.56)
	Amhara	-2.38	-0.24	(-3.64, -1.13)*
	SNNPR	-2.49	-0.25	(-4.00, -0.99)*
	Gambella	-5.75	-0.51	(-8.15, -3.36)*

# continues variable *p < 0.05, ref. =reference

### Healthcare system actors’ SBCC intervention skills

The mean score of overall skill in SBCC intervention was 61.32, with a SD of 25.56, while the standard mean score was 2.36 (SD ± 0.98). More than half (52.6%) of healthcare system actors scored less than the mean score. The standard median score was 2.27, with an IQR of 1.31. Of the sub-domains, the highest median score was observed on implementing and monitoring the change process (median = 2.33 (IQR 1.17)) and the lowest was on evaluating and re-planning (median = 2.14 (IQR 2.00)) (Table 5). The item level skills and competencies score was provided in Supplementary file 2.

**Table 5:** Descriptive statistics of skill in SBCC intervention sub-domains among front line healthcare system actors in Ethiopia, 2020

Variables	Number of items	Mean	SD	Median	IQR	Min	Max
Overall SBCC skill	26	2.36	0.98	2.27	1.31	1	5
Understanding the situation	4	2.41	0.99	2.25	1.25	1	5
Focusing and designing the communication strategy	5	2.39	1.01	2.30	1.20	1	5
Creating interventions and materials for change	4	2.40	0.99	2.25	1.25	1	5
Implementing and Monitoring the Change Process	6	2.39	0.98	2.33	1.17	1	5
Evaluating and Re-planning	7	2.26	1.09	2.14	2.00	1	5

SD: Standard Deviation; IQR: Interquartile Range; Min: Minimum; Max: Maximum

### Correlation of skill with SBCC intervention

A Spearman rank correlation analysis was done to explore the relationship among the sub-domains of skill in the SBCC intervention. Accordingly, all sub-domains of skill in SBCC intervention showed a strong positive and significant relationship among each other at a *p*-value of < 0.05. The overall SBCC skill has a strong and positive association with the sub-domain of SBCC skills at a *p*-value of < 0.05 (Table 6).

**Table 6:** Correlation among subdomains of skill with SBCC among frontline healthcare system actors in Ethiopia, 2020

Variables	(1)	(2)	(3)	(4)	(5)	(6)
(1) Overall SBCC related skills	1.000					
(2) Understanding the situation	0.94*	1.000				
(3) Focusing and designing the communication strategy	0.94*	0.88*	1.000			
(4) Creating interventions and materials for change	0.94*	0.89*	0.90*	1.000		
(5) Implementing and Monitoring the Change Process	0.95*	0.88*	0.87*	0.89*	1.000	
(6) Evaluating and Re-planning	0.93*	0.81*	0.83*	0.83*	0.87*	1.000

### Predictors of skill in SBCC interventions

Place of residence, sex, regional states, age, service year, profession, participation in SBCC-related training, and knowledge of SBCC were fitted in multiple linear regression. In multiple linear regression, sex, service year, profession, region, and knowledge of SBCC were statistically significant variables at a 5% level of significance. The standardized regression coefficient suggested that being from Oromia region (β=-0.68) was the main predictor of skill on SBCC interventions, followed by being from Amhara region β=-0.48) and SNNPR (β= -0.34). The variance explained in skill on SBCC interventions from all predictors was 50.2%.

The analysis showed that male frontline health actors had 5.34 times increased skill in SBCC interventions than females holding other variables constant (β = 5.34, 95% CI (1.14, 9.55)). Health actors who had an experience of 8–11 years had 6.96 times increased skill in SBCC interventions compared to those who had less than or equal to four years’ of experience holding other variables constant (β = 6.96, 95% CI (0.75, 13.17)). Compared to nurses, skill in SBCC interventions decreased by 9.71 times among health officers holding other variables constant (β=-9.71, 95% CI (-15.66, -3.75)). Similarly, frontline health workers from Oromia (β=-40.47, 95% CI (-47.53, -33.42)), Amhara (β=-32.40, 95% CI (-40.07, -24.74)), and SNNPR (β=-21.61, 95% CI (-30.09, -13.14)) regions had 40.47, 32.40, and 21.61 decreased skill in SBCC interventions in comparison to those from Addis Ababa city, respectively while holding other variables constant. Frontline health actors who had good knowledge of SBCC had 9.82 times increased skill in SBCC than those with low knowledge while holding other variables constant (β = 9.82, 95% CI (5.26, 14.38)) (Table 7).

**Table 7:** Multiple linear regression analysis of frontline healthcare system actors’ skill in SBCC interventions in Ethiopia, 2020

Variables		Unstandardized beta(B)	Standardized beta (β)	95% CI of B
Constant		73.95		(66.57,81.33)
Sex	Female (ref)			
	Male	5.34	0.10	(1.14, 9.55)*
Residence	Urban (ref)			
	Rural agrarian	2.42	0.04	(-2.57, 7.41)
	Rural pastoralist	3.35	0.05	(-8.19, 14.89)
Age	≤ 24 years (ref)			
	25–29 years	1.63	0.03	(-4.55, 7.82)
	30–34 years	-4.29	-0.08	(-11.73, 3.14)
	≥ 35 years	-3.10	-0.05	(-12.58, 6.37)
Service year/experience	1–4 years (ref)			
	5–7 years	-1.49	-0.03	(-6.70, 3.72)
	8–11 years	6.96	0.12	(0.75, 13.17)*
	≥ 12 years	4.26	0.07	(-4.71, 13.23)
Profession	Nurse (ref)			
	HEW	-5.50	-0.08	(-11.11, 0.11)
	Health officer	-9.71	-0.15	(-15.66, -3.75)*
	Midwife	-5.53	-0.05	(-12.37, 1.31)
	Others	-0.52	-0.008	(-5.78,4.75)
Ever took SBCC related trainings	No (ref)			
	Yes	11.51	0.22	(6.29, 16.73)
Region	Addis Ababa (ref)			
	Oromia	-40.47	-0.68	(-47.53, -33.42)*
	Amhara	-32.40	-0.48	(-40.07, -24.74)*
	SNNPR	-21.61	-0.34	(-30.09, -13.14)*
	Gambella	-12.70	-0.18	(-26.43, 1.02)
Knowledge on SBCC	Low (ref)			
	Moderate	4.18	0.04	(-1.67, 10.03)
	Good	9.82	0.18	(5.26, 14.38)*

# continues variable *p < 0.05, ref. =reference

## Discussion

In the present study, frontline healthcare system actors’ knowledge and skills in SBCC intervention design, implementation, monitoring and evaluation were assessed. Effective SBCC planning, implementation, and program monitoring and evaluation require healthcare system actors to have all the necessary prerequisite competencies that cut across the field [12]. The study showed that frontline healthcare system actors’ had on average low competencies (knowledge and skills) to plan, implement, and evaluate SBCC interventions. This finding was in line with the study conducted in Malawi, which reported that health promotion officers had low capacity to plan, implement and evaluate SBCC interventions [20].

The mean overall SBCC knowledge score was about 13.2, with a standard deviation of 3.99. Of the SBCC knowledge sub-domains, the highest mean score was observed on understanding the situation and the lowest was on creating interventions and materials for behavior change. The findings of the study revealed that more than half (59.2%) of the participants scored below 60% of the sum score and had overall knowledge of SBCC interventions. Besides, only 35.5% of them reported that they took at least one on the job SBCC or health communication related training in the last five years. This had significant implications on designing and implementing SBCC programs, analyzing the situation and audiences, setting SMART objectives, process of message development, and monitoring and evaluation. This could also influence the standard processes for carrying out SBCC interventions [28]. This finding confirms the need for capacity strengthening in health promotion and SBCC among frontline healthcare actors [20, 29].

In this study, higher service years were linked with an increased overall knowledge score for SBCC. This could be due to a longer service year contributed to engagement in SBCC related initiative and training which contributed to enhanced knowledge about SBCC. On the other hand, frontline healthcare system actors from Amhara, SNNPR, and Gambella regions had decreased knowledge of SBCC compared to those from Addis Ababa city. This could be due to frontline healthcare system actors from Addis Ababa would have improved access to SBCC trainings and programs. This evidence suggested that strengthening capacity of frontline workers with lower service year and in the above mentioned regions to design successful SBCC strategies in Ethiopia.

The present study also found that the standard mean score of overall skill in SBCC intervention was 2.36 (SD ± 0.98). The highest score was observed for implementing and monitoring the change process, while the lowest was on evaluating and re-planning. More than half (52.6%) of healthcare system actors scored less than the mean score. The gap in skills would influence the SBCC program’s effectiveness in changing behaviors by positively influencing knowledge, attitudes, social norms, and the environment. Existing evidence also revealed that skills gaps among health actors hamper SBCC efforts [20]. This depicts that frontline healthcare actors need to be equipped with robust tools and skills to deal with existing and emerging challenges. For instance, the rapidly changing environment and increasing need for policy convergence require continuous capacity building for health promotion professionals [1, 29]. Thus, SBCC interventions also need a plan for strengthening frontline actors’ SBCC capacity through different approaches. Continuous capacity building and refreshment training are proven approaches to increasing SBCC capacity and helping individuals further sharpen their knowledge and skills [28].

The current study also found that good knowledge, being male, and having experience of 8–11 years were linked with improved skill in SBCC. Frontline healthcare system actors possessing good knowledge in the areas of design, implementation, monitoring, and evaluation of SBCC interventions may be better equipped to carry out SBCC interventions. Besides a longer service year contributed to enhanced exposure to SBCC initiative and develop knowledge about SBCC. On the other hand, it was found that health officers and frontline healthcare system actors from Oromia, Amhara, and SNNPR regions had reduced skill in SBCC. This may be due to the fact that in primary healthcare setting, nurses are the primary healthcare system actors in disseminating health messages, and health officers concentrate on delivering curative service. This discrepancy could be attributed to differences in access to SBCC-related training among healthcare system actors and regions. In addition, healthcare system actors in Addis Ababa have wide opportunities to engage in SBCC programs and capacity building opportunities that may have positively contributed to increased skills in SBCC. This finding articulate that the capacity building activities to enhance SBCC skill need to consider frontline healthcare actor’s knowledge, service year, and regional variation.

The SBCC environment in Ethiopia needs to be capable of continuing, improving on, and adapting effective SBCC efforts. The expansion of SBCC interventions in the country demands human resources with specific skills and competencies. The national strategy also pointed out the lack of knowledge and skills of frontline healthcare actors as a challenge. This may not only be attributed to a gap in individual capacity rather, lack of organizational plans for SBCC efforts could be reflected in knowledge and skill limitations. Besides, the SBCC’s efforts need to approach issues at multiple levels of society, have local ownership and be responsive to changing needs. This calls for further strengthening the capacity of the frontline healthcare system actors. The need for capacity strengthening in SBCC health actors was well-documented in previous evidence [3, 20, 30]. Thus, in-service training for healthcare system actors should emphasize SBCC contents, the fundamental pillar of public health interventions. In addition, on-the-job training and continued support and mentoring contribute to the improved knowledge and skills that had been imparted 31.

### Strength and limitations

To the best of our knowledge, this is the first study assessing frontline actors’ knowledge and skills in designing, implementing, monitoring, and evaluating SBCC interventions in Ethiopia. The assessment tool was also powerful in that it established the baseline of the frontline healthcare system actor’s knowledge and skill in SBCC and conducive to develop tailored interventions. This study is not free from limitations. A large portion of the variance in SBCC knowledge is not explained by the regression model. Future studies must therefore take into account more explanatory variables. Due to the cross-sectional nature of the study, causal inference is difficult between the independent and dependent variables. Social desirability bias might overestimate the participant’s knowledge and skill. In addition, the frontline health worker’s SBCC skills were assessed based on the respondents’ self-reports. In order to lessen social desirability bias, the questionnaire was self-administered and the questions were properly constructed.

## Conclusion

The overall knowledge and skill of frontline healthcare system actors on SBCC in Ethiopia was low. The SBCC knowledge was significantly predicted by variation in the service year and the regional states. Whereas, sex, service year, profession, regional variation, and knowledge of SBCC were predictors of SBCC skill. The regression model best suited and explained half of the variance in SBCC skill, however it was only weekly fitted with SBCC knowledge. Providing adequate and ongoing on-the-job SBCC training for frontline healthcare system actors is essential to improve their SBCC knowledge and skill. The capacity building trainings for SBCC actors need to consider on regional variation, females, health officers, those with low service years, and those with low knowledge for the ultimate improvement of health outcomes. Specific domains of SBCC knowledge and skills need special attention at different levels in the process of SBCC capacity strengthening.

### Electronic supplementary material

Below is the link to the electronic supplementary material.

Supplementary Material 1

## Data Availability

The datasets analyzed in the current study are publicly available from the corresponding author (Simegnew Handebo) on reasonable request by simegnewh@gmail.com.

## Abbreviations

BCC: Behavior Change Communication
C4D: Communication for Development
CI: Confidence Interval
EMDHS: Ethiopian mini demographic and health survey
HEWs: Health Extension Workers
IEC: Information Education and Communication
MOH: Ministry of Health
RHB: Regional Health Bureau
SBCC: Social and Behavior Change Communication
SD: Standard Deviation
UNICEF: United Nations Children’s Fund
USAID: Agency for International Development
WHO: World Health Organization
