# Supplementary material for: Social and behavior change communication competency among front-line healthcare system actors in Ethiopia: a cross-sectional study
Source: BMC Public Health. 2024 Mar 1;24:663. doi: 10.1186/s12889-024-18084-x (PMC10908142; doi:10.1186/s12889-024-18084-x)
Supplement: Supplementary file 1 — Supplementary Material 1 [file 12889_2024_18084_MOESM1_ESM.docx]

**Supplementary 1: Items score of health system actors’ knowledge on Social and Behavior Change Communication**

| **S.no** | **Knowledge Items** | **Frequency (%)** | |
| --- | --- | --- | --- |
|  |  | **Incorrect** | **Correct** |
| **Section 0: Basic SBCC Concepts** | | | |
| K1 | From which level does a socio‐ecological model address change? | 110 (22.5) | 378(77.5) |
| K2 | Which of the following is NOT a cross‐cutting principle of SBCC? | 270 (55.3) | 218 (44.7) |
| K3 | Which of the following is a key characteristic of SBCC? | 107 (21.9) | 381 (78.1) |
| **Section 1: Understanding the Situation** | | | |
| K4 | People who have worked on a topic for many years can skip the step called Understanding the Situation. | 299 (61.3) | 189 (38.7) |
| K5 | Which of the following applies to SBCC theories and theoretical models? | 291 (59.6) | 197 (40.4) |
| K6 | Which of the following is addressed when an SBCC program uses a socio‐ecological model? | 130 (26.6) | 358 (73.4) |
| K7 | Checking assumptions by looking at existing research is a key step in understanding the situation. | 133 (27.3) | 355 (72.7) |
| K8 | Which of the following is a reason to review stakeholder activities during a situational analysis? | 160 (32.8) | 328 (67.2) |
| **Section 2: Focusing and Designing the Communication Strategy** | | | |
| K9 | Which of the following does NOT describe the role played by a SBCC communication strategy? | 218 (44.7) | 270 (55.3) |
| K10 | For a family planning program for urban men and women of reproductive age, which of the following groups in a people analysis would be considered among the people directly influencing the people most affected? | 301 (61.7) | 187 (38.3) |
| K11 | Communication objectives are audience specific, measurable, and what else? | 277 (56.8) | 211 (43.2) |
| K12 | A strategic approach refers to the way an SBCC program decides to package activities into a single intervention, campaign, or activity. | 220 (45.1) | 268 (54.9) |
| K13 | SBCC activities will have the greatest impact if which of the following is done? | 273 (55.9) | 215 (44.1) |
| **Section 3: Creating Interventions and Materials for Change** | | | |
| K14 | Which of the following is generally NOT part of a creative brief? | 305 (62.5) | 183 (37.5) |
| K15 | Which if the following is NOT a key element of material and message design? | 372 (76.2) | 116 (23.8) |
| K16 | There is no real difference between a concept test and a pretest. | 205 (42.0) | 283 (58.0) |
| K17 | Which of the following should never come last when developing SBCC materials? | 387 (79.3) | 101 (20.7) |
| **Section 4: Implementing and Monitoring the Change Process** | | | |
| K18 | Which of the following is NOT a major component of an SBCC program work plan? | 441 (90.4) | 47 (9.6) |
| K19 | What is a major expense category that should be included in an SBCC budget? | 141 (28.9) | 347 (71.1) |
| K20 | Which of the following is the most important for staff managing and implementing an SBCC program? | 207 (42.4) | 281 (57.6) |
| K21 | Which of the following should be included in the supervision of SBCC field workers and volunteers? | 97 (19.9) | 391 (80.1) |
| **Section 5: Evaluating and Re-planning** | | | |
| K22 | Output and quality are two types of monitoring indicators. | 120 (24.6) | 368 (75.4) |
| K23 | Which of the following is NOT a key decision to be made before data collection? | 301 (61.7) | 187 (38.3) |
| K24 | Which of the following is NOT an example of a typical monitoring tool? | 345 (70.7) | 143 (29.3) |
| K25 | Which of the following is typically NOT included in an M&E plan? | 289 (59.2) | 199 (40.8) |
| K26 | You only need monitoring data for re‐planning | 253 (51.8) | 1. (48.2) |

**Supplementary 2: Items score of health system actors’ skills and competencies on Social and Behavior Change Communication**

| **S. No** | **Skills and Competencies Items** | **Mean** | **Standard deviation** |
| --- | --- | --- | --- |
| **Section 1: Understanding the Context through Situation Analysis** | | | |
|  | Conducting a situation analysis | 2.43 | 1.06 |
|  | Using theories or models for situation analysis or communication strategy design | 2.39 | 1.09 |
|  | Using research data to assist with SBCC program design. | 2.38 | 1.09 |
|  | Reviewing activities of stakeholders during a situation analysis to reduce replication of programs. | 2.45 | 1.07 |
| **Section 2: Focusing and Designing the Communication Strategy** | | | |
|  | Developing a communication strategy | 2.37 | 1.14 |
|  | Conducting audience segmentation | 2.51 | 1.09 |
|  | Developing communication objectives | 2.43 | 1.08 |
|  | Determining an effective channel mix to reach audiences | 2.40 | 1.10 |
|  | Using a strategic approach that links all strategies and channels into a coordinated effort | 2.24 | 1.18 |
| **Section 3: Creating Interventions and Materials for Change** | | | |
|  | Developing creative briefs | 2.57 | 1.11 |
|  | Conducting concept testing with key audiences | 2.51 | 1.08 |
|  | Conducting stakeholder and technical reviews of messages and developed materials | 2.24 | 1.17 |
|  | Pretesting materials with key audiences | 2.31 | 1.14 |
| **Section 4: Implementing and Monitoring Change Processes** | | | |
|  | Developing a detailed work plan | 2.51 | 1.04 |
|  | Coordinating implementation with other SBCC programs | 2.38 | 1.04 |
|  | Developing detailed and accurate budgets before initiating SBCC activities | 2.39 | 1.07 |
|  | Coordinating implementation of activities for impact (i.e., considering competing activities, synchronizing program elements, having activities enhance each other) | 2.34 | 1.08 |
|  | Determining if management and staff have the capacity to manage and implement SBCC programs | 2.26 | 1.15 |
|  | Providing regular supervision of field staff and ensuring they have materials available (including through field observation) | 2.49 | 1.09 |
| **Section 5: Evaluating and Re-planning** | | | |
|  | Developing M&E plans for SBCC programs | 2.26 | 1.12 |
|  | Developing indicators for SBCC programs that are linked with communication objectives | 2.27 | 1.16 |
|  | Developing tools to monitor implementation of SBCC programs | 2.25 | 1.15 |
|  | Putting a system in place to assure high quality of M&E data collection and analysis | 2.24 | 1.17 |
|  | Documenting and disseminating results, lessons learned, and best practices | 2.24 | 1.15 |
|  | Analyzing and sharing M&E data with implementers of SBCC programs | 2.27 | 1.17 |
|  | Using M&E data to improve current SBCC programs | 2.26 | 1.19 |
|  | Overall SBCC Skills standard score | 2.36 | 0.98 |
